# Alveolar Soft Part Sarcoma of the Nasolabial Fold: A Case Report and Literature Review

**DOI:** 10.7759/cureus.38310

**Published:** 2023-04-29

**Authors:** Tristan M Palmer, Westin M Yu, Jamie M Moenster

**Affiliations:** 1 Surgery, Lake Erie College of Osteopathic Medicine, Erie, USA; 2 Plastics, Dermatology and Plastic Surgery of Arizona, Tucson, USA

**Keywords:** chemotherapy, sarcoma, next generation sequencing (ngs), otolaryngology, intraoral excision, plastic and reconstructive surgery, immunohistochemistry staining, nasolabial fold, aspscr1-tfe3, alveolar soft part sarcoma

## Abstract

Alveolar soft part sarcoma (ASPS) is a rare malignancy that is morphologically characterized by a distinctive nodular, organoid, or nested growth pattern in which the cells are separated by vascularized septa. The diagnosis is based on a combination of pathologic and immunohistochemical findings and the presence of an *ASPSCR1-TFE3* gene fusion revealed by next-generation sequencing. ASPS most commonly occurs as a painless mass in the lower extremity, with likely involvement in the lungs if metastasis is present. Here we report a case of ASPS that exhibited the characteristic *ASPSCR1-TFE3* gene fusion along with a reciprocal fusion of *TFE3-ASPSCR1,* which presented in the nasolabial fold of a 31-year-old female. An intraoral approach was utilized for complete surgical resection of the malignancy, resulting in continued remission after 11 months.

## Introduction

Alveolar soft part sarcoma (ASPS) is a rare, slow-growing malignancy that accounts for approximately one percent of all sarcomas and 80 new diagnoses per year [1]. ASPS is typically found in younger patients, between the ages of 15 and 35, with a slight female predilection [1,3]. The most common site of occurrence is the extremities, with the lower extremity demonstrating the highest prevalence [4,5]. One of the most distinguishing characteristics of ASPS is an *ASPSCR1-TFE3* gene fusion, with *ASPSCR1* located on chromosome 17 and the *TFE3* gene on the X chromosome, thus accounting for female predilection. ASPS is characterized as a delayed, metastasizing tumor that most commonly spreads to the brain and lungs decades after diagnosis, with a higher five-year survival rate in patients without metastasis. To date, there have been fewer than 20 reported cases of ASPS occurring in the buccal/oral region in literature. We report a case of ASPS with the characteristic *ASPSCR1-TFE3* gene fusion and positive tumor markers presenting in the nasolabial region of a 31-year-old female patient.

## Case presentation

**Figure 1:**
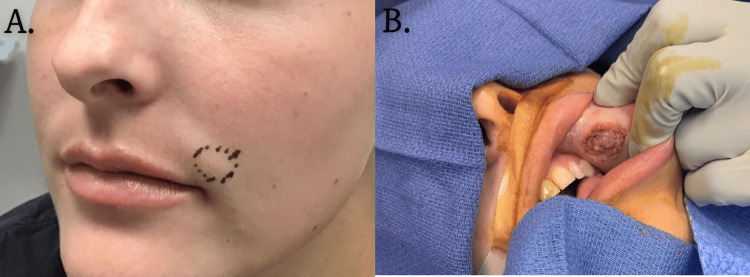
Clinical presentation A. Pre-operative mass located in the left nasolabial fold. B. Intra-oral surgical findings demonstrated a mass with a pink-tan, wrinkled mucosal surface that contained a curvilinear crevice.

A 31-year-old female presented to the plastic surgery office for the excision of a mass located in her left nasolabial fold (Figure 1). The patient noted increasing enlargement of the mass over 12 months, with a complaint of painful smiling and yawning. Additionally, the patient's history indicated an absence of notable risk factors for developing ASPS, such as a family history of sarcoma, Li-Fraumeni syndrome, radiation, chemical, or occupational exposures.

Based on the location and scar consideration, an intraoral approach was utilized for excision under general anesthesia per patient preference. Preoperative imaging and biopsy were not obtained for this mass as it was originally believed to be a benign cyst. The preoperative size of the lesion measured 2.3 cm x 2.3 cm, and a margin of 0.2 cm was taken on all sides. Grossly, the mass was described as a raised, flesh-colored papulo-nodule that exhibited a pink-tan, wrinkled mucosal surface with a curvilinear crevice (Figure 1). An intermediate repair was performed to minimize tension and achieve optimal functional and cosmetic results. The soft tissue mass was then sent to pathology for analysis, and consultation from an outside institution was obtained. Additionally, the patient was evaluated by the department of otolaryngology-head and neck surgery at an outside medical center, and re-excision was performed to assess for remaining malignancy, which came back negative. Re-excision was performed due to inadequate tumor resection margins, as the original operation was initially indicated for cyst removal.

**Figure 2:**
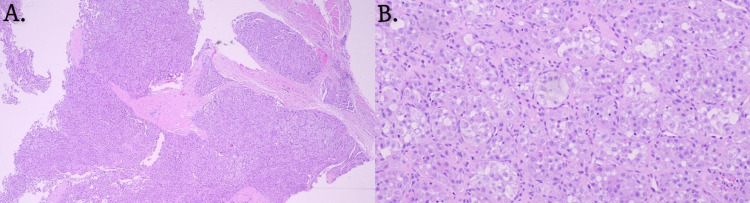
Histological images stained with hematoxylin and eosin A. 40x low-power magnification of the alveolar soft part sarcoma (ASPS) excised from the nasolabial fold. B. 100x medium-power magnification demonstrating the presence of polygonal cells with eosinophilic cytoplasm in a nested growth pattern that is separated by vascular fibrous septa.

The pathology report detailed histologic features demonstrating a malignant epithelioid neoplasm with a lobulated and vaguely nested growth pattern (Figure 2). Cytologically, large and mostly uniform cells with prominent nucleoli and round to oval vesicular nuclei were found, in combination with abundant eosinophilic cytoplasm and variable clearing. Intracytoplasmic crystal-like structures were discovered in a subset of cells, but no tumor necrosis was identified. The differential diagnosis included perivascular epithelioid cell tumor (PEComa), melanoma, and metastatic renal cell carcinoma, but the immunohistochemical (IHC) profile and molecular findings argued against these possibilities. The tumor was found to be IHC positive for smooth muscle actin (SMA), desmin, E-cadherin, and vimentin but negative for pan-keratin, cytokeratin 7 (CK7), cytokeratin 20 (CK20), epithelial membrane antigen (EMA), S100, melanoma-associated antigen recognized by T cells (Mart-1), GATA3, estrogen receptor (ER), gross cystic disease fluid protein 15 (GCDFP15), tumor protein 63 (p63), carcinoembryonic antigen (CEA) and CD15. The tumor was also positive for periodic acid-Schiff (PAS) and PAS diastase stains. Next-generation sequencing sarcoma gene fusion panel was performed, which demonstrated a fusion of *ASPSCR1* on exon 7 and *TFE3* on exon 5, along with a reciprocal fusion of *TFE3-ASPSCR1* between *TFE3 *on exon 3 and *ASPSCR1* on exon 8. Cumulatively, the tumor’s histology, immunohistochemical staining, and molecular results confirmed the exceptionally rare diagnosis of ASPS.

Follow-up care included an appointment every six months to monitor re-occurrence and metastasis, as well as a presentation of the case to the sarcoma board, leading to the decision that the patient is unlikely to require systemic therapy. At the last follow-up, six months after the operation, no re-occurrence or metastasis was detected on positron emission tomography (PET). The current follow-up protocol for this patient includes yearly PET scans as well as clinical re-evaluation every six months for the first three years, then subsequent yearly evaluations as suggested by the multidisciplinary tumor board.

## Discussion

We report a case of alveolar soft part sarcoma (ASPS) of the nasolabial fold in a 31-year-old female. ASPS is an infrequent type of soft tissue sarcoma (STS), comprising less than 0.0001% of new adult malignancies [1,2]. ASPS most commonly presents as a slow-growing, painless mass located in the lower extremity in young adults and exhibits a slight female predilection [3-5]. Hagerty et al. identified 293 patients diagnosed with ASPS and found the primary site of occurrence to be in the lower extremity, which accounted for 51.9% of cases, while trunk and head/neck comprised 27.3% and 3.4%, respectively [4].

There have been fewer than 20 reported cases of ASPS in the buccal/oral region, with the lingual region being the most frequently observed location. As a result, its occurrence in the nasolabial fold is a remarkably unique presentation of ASPS [4]. Furthermore, ASPS in the head and neck region most frequently presents in the third decade of life, with a median age of occurrence of 25 years old, indicating that our patient is older than previous cases described [6-9]. The combination of the irregular location and older age of presentation in our patient further demonstrates the exceptional nature of this tumor occurrence. Irrespectively, patients with primary head and neck tumors were found to present at diagnosis with a significantly decreased propensity for metastasis than those located in the extremities (40% and 73%, respectively) [4]. A plausible explanation for this occurrence is increased visibility and functional impairment of the malignancy, thus leading to an earlier seeking of treatment. As noted in our case, the patient’s chief complaint was increased pain upon engagement of a growing mass. Thus, it is imperative that physicians remain vigilant in evaluating all masses, as a seemingly benign cyst or mass may conceal malignancy. Regardless of the primary tumor site, ASPS metastasis is most commonly identified in the lung, with the brain and the bone also being reported, and rarely demonstrates regional lymph node spread [3,4].

The five-year survival rate for ASPS is estimated at 86% for those without metastatic spread and 62% for patients reported with metastasis [10]. Metastasis is the main predictor of prognosis, with age being a secondary contributing factor as older individuals are more susceptible to metastasis [1,3,4,10,11]. Other predictors of a worse prognosis in ASPS include male sex, tumor size greater than 5 cm in local lesions, 10 cm in metastatic lesions, and a primary site other than the extremity [1,4,10]. Recurrence of ASPS after resection is a known complication of this malignancy; Wang et al. reported an estimated overall local recurrence rate of 38.9%, with a 33% recurrence rate in their observational study. Careful monitoring is needed in all patients after diagnosis and resection of ASPS, in addition to an individualized schedule based on metastasis and risk factors [3].

Grossly, ASPS is usually characterized by a well-circumscribed lesion that has a soft and rubbery consistency and can vary in color from yellow to gray to tan; areas of necrosis and hemorrhage can also be demonstrated [12,13]. The size of ASPS can range in presentation from 1.2 cm to 24 cm, with a mean size of 6.5 cm, and variability depends on the primary tumor site [9]. This morphology was evident in our patient, as the gross pathological report described the well-circumscribed tumor as containing multiple segments of pink to tan, slightly rubbery soft tissue that measured 2.0 cm. Histologically, ASPS is characterized by a distinctive nodular, organoid, or nested growth pattern where the nested cells are generally consistent in both size and shape. The cells are separated by vascularized septa lined by endothelial cells, and focal necrosis may also be seen in the center of the nests with a pseudovascular pattern. The cytoplasm of these cells can be described as eosinophilic, with a clear appearance with uniform and centrally located nuclei [12-14]. A notable characteristic feature of this tumor is also a PAS and diastase-resistant rhomboid-shaped crystal, which is reported in up to 80% of cases [13].

The aforementioned histologic patterns were observed in our patient, as the pathology report described the malignant epithelioid neoplasm as being lobulated with vaguely nested growth patterns. Cytologically, the cells were described as large, mostly uniform, with round to oval vesicular nuclei, prominent nucleoli, and abundant eosinophilic cytoplasm with variable clearing (Figure 2). Some intracytoplasmic crystal-like structures were also seen in a subset of cells. Immunohistochemically, ASPS can be positive for periodic acid-Schiff (PAS), vimentin, myoglobin, CD34, and CD31, desmin, S-100, cytokeratin, Human Melanoma Black 45, and smooth muscle actin [11-14]. Our patient was positive for PAS, PAS diastase, SMA, desmin, E-cadherin, and vimentin and negative for Pan-keratin, CK7, CK20, EMA, S-100, MART1, GATA3, ER, GCFP15, P63, CEA, and CD15.

Currently, complete surgical resection is the first-line treatment for ASPS, as it offers the greatest benefit [4,10,15]. ASPS is characteristically resistant to chemotherapy, as a less than 10% response rate to conventional anthracycline-based chemotherapy has been noted [16,17]. Currently, chemotherapy is not indicated for ASPS as it has not shown adequate efficacy, but further research is needed as a possible treatment strategy in the future [16]. Radiotherapy is currently used for incomplete surgical resection or when the surgical margin is questionable and is currently being evaluated for a role in preventing local recurrence [16].

Targeted therapy of ASPS is showing increased success with tyrosine kinase inhibitors, specifically sunitinib and pazopanib, which are currently recommended by the National Comprehensive Cancer Network (NCCN) and the Chinese Society of Clinical Oncology (CSCO) [1,4,16,17]. Anlotinib, a novel tyrosine kinase inhibitor that targets vascular endothelial growth factor (VEGF)/ vascular endothelial growth factor receptor (VEGFR) signaling, is recommended for the first-line therapy of ASPS by the CSCO [16]. Furthermore, immune therapy is an area of increasing research, as pembrolizumab and, more recently, atezolizumab have demonstrated effectiveness in patients with unresectable or metastatic ASPS [16]. It is important to note that atezolizumab and pazopanib are the only FDA-approved agents that have demonstrated effectiveness in metastatic ASPS [15,18]. Atezolizumab achieves a therapeutic effect by targeting the programmed cell death ligand 1 (PD-L1) checkpoint protein on tumor cells, while pazopanib acts by inhibiting an intracellular tyrosine kinase of the vascular endothelial growth factor receptor and platelet-derived growth factor receptor [15-18]. Combination therapy for ASPS is also an increasing avenue of treatment currently being explored by researchers [15-17]. However, further research is needed to improve the pharmacological treatment of ASPS while complete surgical resection continues to be the mainstay therapy.

## Conclusions

Alveolar soft part sarcoma (ASPS) is an exceedingly rare type of sarcoma that has less than 20 reported incidences in the buccal/oral region in the literature. Herein, we report a case of ASPS that presented in the left nasolabial fold in a 31-year-old female that exhibited the characteristic *ASPSCR1-TFE3* gene fusion along with a reciprocal fusion of *TFE3-ASPSCR1* between *TFE3* on exon 3 and *ASPSCR1* on exon 8. The patient underwent complete surgical resection of the malignant tumor and has been in remission for 11 months. The current follow-up protocol includes yearly PET scans and clinical re-evaluation every six months for the first three years, then subsequent yearly evaluations to monitor for the presence of local recurrence or metastasis. As a consequence of the uncharacteristic location and age presentation that this exceptionally rare tumor demonstrated, increased clinical suspicion is warranted for a well-circumscribed mass of an unknown etiology presenting in an otherwise healthy patient. This case report and literature review demonstrate the importance of why surgeons need to be cognizant of malignancy when evaluating even the most seemingly benign masses.
